# Chitosan-Based Aerogel Particles as Highly Effective Local Hemostatic Agents. Production Process and In Vivo Evaluations

**DOI:** 10.3390/polym12092055

**Published:** 2020-09-10

**Authors:** Daria Lovskaya, Natalia Menshutina, Maria Mochalova, Artem Nosov, Alexander Grebenyuk

**Affiliations:** 1 International Center for transfer of Pharmaceutical and Biotechnology, Mendeleev University of Chemical Technology of Russia, 125047 Moscow, Russia; chemcom@muctr.ru (N.M.); mochalovamarie@yandex.ru (M.M.); 2 Department of Pharmaceutical Chemistry, Saint Petersburg State Chemical Pharmaceutical University, 197376 Saint Petersburg, Russia; artem_svu06@mail.ru (A.N.); grebenyuk@spmt.ru (A.G.)

**Keywords:** aerogels, chitosan, blood sorption, in vivo evaluations, local hemostatic agent

## Abstract

Chitosan aerogels with potential applications as effective local hemostatic agents were prepared using supercritical carbon dioxide drying to preserve the chitosan network structure featuring high internal surfaces and porosities of up to 300 m²/g and 98%, respectively. For the first time, hemostatic efficacy of chitosan-based aerogel particles was studied in vivo on a model of damage of a large vessel in the deep wound. Pigs were used as test animals. It was shown that primary hemostasis was achieved, there were no signs of rebleeding and aerogel particles were tightly fixed to the walls of the wound canal. A dense clot was formed inside the wound (at the femoral artery), which indicates stable hemostasis. This study demonstrated that chitosan-based aerogel particles have a high sorption capacity and are highly effective as local hemostatic agents which can be used to stop massive bleeding.

## 1. Introduction

**Figure 1:**
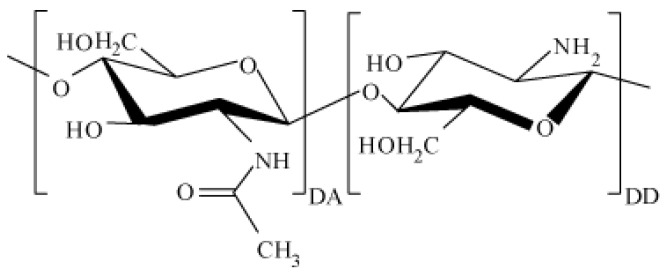
The structural formula of chitosan.

According to statistics from the World Health Organization, the main cause of death in injuries is a large amount of blood loss. The development of new functional materials for the creation of highly effective local hemostatic agents is an urgent task for modern science. Aerogels based on various biopolymers that have such properties as large specific surface area, high porosity and sorption capacity, low density and biological compatibility with human tissues and organs [1,2] are gaining increasing interest. Chitosan biopolymer is a natural polysaccharide that is completely safe for humans; it has antimicrobial activity and is able to absorb biological fluids, including blood [3,4]. Chitosan macromolecules consist of randomly bound β-(1-4)-D-glucoseamine units and *N*-acetyl-D-glucosamine (Figure 1).

Chitosan is obtained by deacylation of chitin, which is located in the cell walls of fungal cells, crustacean shells and insects. To date, a high level of purification of chitosan from various impurities (up to 85%) has been achieved. However, chitosan processed from chitin may contain various heavy metals, protein residues and others [5]. In the case of biomedical applications, those impurities can have an impact on humans and have to be considered when creating and registering a new medical product.

Due to the large number of amino groups, the chitosan molecule can acquire an excess positive charge, bind hydrogen ions, various metal ions [6], as well as water-soluble substances (e.g., bacterial toxins and toxins formed during digestion via hydrogen, dipol–dipol or ionic bonding). Chitosan is the second most common polysaccharide derived from biomass, while it has a fairly low cost and is an environmentally friendly product. The listed features of chitosan allow it to be of great use in medicine, biotechnology and bioengineering. For example, biocompatible protective coatings for artificial bioprostheses, including artificial heart valves, can be made from thin films based on chitosan [7]. Chitosan-based films can be used as drug delivery systems through the mucous membranes on the surface of the eyes [8]; it is possible to create scaffolds for regeneration and growth of bone cells, since chitosan is known to promote cell proliferation and further bone mineralization [9]. In medicine and pharmaceuticals, chitosan is often used in the form of hydrogel. Some studies show that applying N-carboxybutyl chitosan directly to the affected area helps in wound healing and reduces scar formation after plastic surgery. Other studies provide information that applying chitosan ascorbate directly to the gums helps in the treatment of periodontitis.

Additionally, aerogels based on chitosan have a large specific surface area (149–739 m^2^/g) [10]. Considering the process of obtaining chitosan aerogels, various factors can affect the structural and physicochemical characteristics of the final material. Such factors include the pH of the reaction medium, the temperature and duration of the reactions, the concentration of the initial materials, the nature and concentration of the catalysts and much more [11]. For example, insufficient reaction time can lead to the crosslinking agent not fully reacting with all amino groups [12], which can affect the structure of the aerogel. In [13], it is shown how, by changing parameters such as pH of the medium and ion concentration, the density and specific surface area of the aerogel can be changed. In particular, in this work, it was noted that lowering the pH of the medium increases specific surface area, porosity and strength of the aerogel skeletal structure. In [14], it is mentioned that varying the properties of the original biopolymer (molecular weight, composition, degree of branching of the biopolymer) has a significant effect on both the bulk properties of aerogels and porosity. In particular, it was shown in [15] that the porosity of the obtained aerogel decreases from 90% to 76% with a simultaneous increase in the concentration and molecular weight of the polymer. One of the parameters that may possibly affect the structural and physicochemical characteristics of chitosan aerogel is the molecular weight. Frequently in literature, the influence of molecular weight on the bioactivity of the resulting materials is considered. In [16], it is shown that the hemostatic activity of chitosan increases with molecular weights in the range of 50–190 kD and an increase in the degree of deacylation to 88%. Chitosan salts such as lactate and acetate also have high hemostatic activity (ability to stop bleeding). This is associated with an increase in solubility. Due to the increase in the molecular weight of chitosan, its antibacterial properties are enhanced, and the use of substituted chitosan promotes an increase in cytotoxicity [16,17]. As a result of studies carried out in [16], it was revealed that chitosan oligomers have a lower antibacterial effect than chitosan itself, while inhibiting the growth of various bacteria differed for polymers with different molecular weights.

If bleeding stops, the rate of blood clot formation, as well as the aggregation of its shaped elements, is associated with an increase in the specific surface area and porosity of chitosan particles [18,19], which creates a great prospect for the use of chitosan-based aerogels as local hemostatic agents. When placed in a wound, chitosan aerogel can significantly absorb wound exudate and turn into a hydrogel, which completely fills the wound, preventing the formation of exudate-filled areas that are a favorable environment for the growth of bacteria. In particular, in [16], aerogel particles from chitin and graphene were developed, which were used as sorbents of bilirubin from human blood, since liver function deteriorates with an excessive concentration of this compound in the blood. In addition, these aerogel particles showed a low ability to destroy red blood cells and increase the overall anticoagulability in the blood.

Based on this review, it can be assumed that biopolymer aerogels based on chitosan are able to integrate the unique physicochemical and structural characteristics of aerogels in combination with the natural properties of chitosan. Due to their properties, chitosan aerogels seem to be relevant materials for stopping bleeding of different natures, including massive venous and arterial bleeding. At present, in the modern scientific and technical literature, comprehensive data on the use of chitosan aerogels as local hemostatic agents are not presented, which makes the present studies relevant. In the framework of this work, a comprehensive study of the process of obtaining particles of chitosan aerogels was carried out, the data of in vivo studies of hemostatic efficacy on laboratory animals were first presented.

## 2. Materials and Methods

### 2.1. Synthesis of Chitosan-Based Gel Particles via Dripping Method

**Figure 2:**
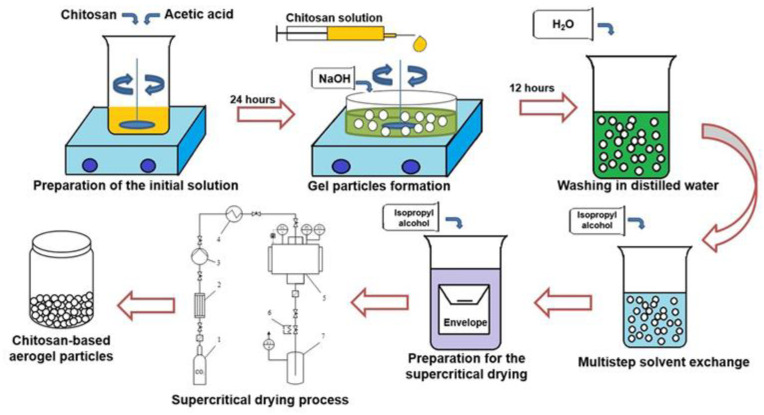
Scheme of the synthesis of chitosan-based aerogel particles.

To obtain chitosan-based gel particles, the dripping method was used [20]. The overall scheme of the process for synthesis of chitosan-based aerogel particles is presented in Figure 2.

To prepare the initial solutions, chitosan with different molecular weight (Sigma-Aldrich, Saint Louis, MO, USA) and chemically pure acetic acid (Sigma-Aldrich) was used. The molecular weight of the chitosan was 111, 125, 294, 343 kD, respectively. To prepare the solutions, a certain amount of chitosan was mixed with acetic acid solution (0.1 M) using a magnetic stirrer to obtain the desired concentration of chitosan solution (1 wt.%). Mixing was continued for 24 h to ensure complete dissolution. Chemically pure sodium hydroxide (Sigma-Aldrich) was used as a crosslinking agent for the formation of a gel via the dripping method. To prepare the solution of crosslinking agent, a certain amount of sodium hydroxide was mixed in distilled water using a magnetic stirrer in order to obtain a 4 molar solution. The chitosan solution was added drop-wise through a needle into the solution containing the crosslinking agent using a piston pump (with constant stirring). The flow rate was 1 mL/min. The distance from the needle to the surface of the crosslinking agent was 20 mm (the distance was chosen so that round particles were formed during the process). The process of gel particle formation via the dripping method consists of two main stages: the formation of droplets of the initial solution by dispersion and gelation, which occurs when the droplets enter the liquid containing the dissolved cross-linking agent. Gelation occurs due to the fact that the sodium hydroxide diffuses into droplets and polymer crosslinking occurs. As a result, gel particles with a diameter of 2–5 mm formed. The obtained gel particles were left in the solution of sodium hydroxide for 12 h (in order to be sure that all chemical reactions were completed). Then, the pH of the gel particles was adjusted to neutral by repeated washing in distilled water. The next step was the multistep solvent exchange (in this work, the isopropyl alcohol was used). At each step, the concentration of isopropyl alcohol increased. In this work, the following solvent exchange steps were used: 10%, 30%, 60%, 90%, 100%, 100%. At least 2 h must elapse between each step. Multistep solvent exchange is necessary in order to maintain the original gel structure, avoiding shrinkage and cracking, which will negatively affect the quality of the final aerogel particles. The final stage is supercritical drying, which was carried out similarly to [20,21]. Supercritical drying is considered as the most important step since it enables the preservation of the three-dimensional pore structure which leads to the unique properties of the aerogel (high porosity and large surface area). In this work, carbon dioxide was used as the supercritical fluid. Process parameters: temperature 40 °C, pressure 12–14 MPa, carbon dioxide consumption 0.2 kg/h. Drying time was 6 h.

### 2.2. Analytical Experiments

The textural characterization of the obtained chitosan-based aerogel particles was carried out by low-temperature N_2_ adsorption–desorption analysis (ASAP 2020MP, Micromeritics, Norcross, GA, USA). Before the measurements, samples were dried under a vacuum at 50 °C for 20 h. Specific surface area was determined by the BET method. BJH analysis was used to determine the average pore diameter of aerogel particles using desorption techniques. Aerogel shape and appearance were analyzed using SEM (JEOL 1610LV, JEOL Ltd, Tokyo, Japan). Samples were platinum-sputtered prior to imaging in order to minimize charging and improve the image contrast. The skeletal density was determined by the pycnometer (AccuPyc II 1340 helium pycnometer, Micromeritics, Norcross, GA, USA). The bulk density was determined as the ratio of particle mass to volume. The density of the aerogel particle was calculated using the volume of one single particle to the mass of 20 particles. The porosity of the aerogel particles was calculated based on skeletal and overall density. Analytical experiments were performed at the core facilities centre of Mendeleev University of Chemical Technology of Russia. The sorption capacity of chitosan-based aerogel particles was measured using distilled water; for this, a given number of particles was taken, placed in a certain volume of water, exceeding the particle volume by at least 80% and kept for 30 min. Measurements were repeated 3 times. The average sorption capacity was determined as the ratio of the mass of water in the pores of the aerogel to the mass of the aerogel.

### 2.3. In Vivo Evaluations

In the framework of this work, in vivo studies were performed on laboratory animals (pigs). The study was approved by the independent Ethical committee of the Federal State Budgetary Educational Institution of Higher Education «Military Medical Academy named after S.M. Kirov» Ministry of Defense of the Russian Federation (Approval number 199 dated 19 December 2017). Two types of chitosan were used: chitosan powder intended to stop bleeding (hereinafter «Chitosan») and chitosan-based aerogel particles obtained from chitosan with a molecular weight of 111 kD (hereinafter «Chitosan aerogel»). Both chitosan samples were placed in a plastic applicator (in order to conveniently place the hemostatic agent in the wound).

The studies were conducted on 6 pigs «large white» with a weight of 38.5–44.5 kg, in accordance with the requirements of regulatory documents on the procedure for conducting experimental work with animals (Directive 2010/63/EU of the European Parliament and of the Council of the European Union of 22.09.2010 on the protection of animals used for scientific purposes). In the vivarium, the animals were on a normal diet. On the day of the experiment, the animals were anesthetized and pharmacologically prepared according to the protocol described in [22].

**Figure 3:**
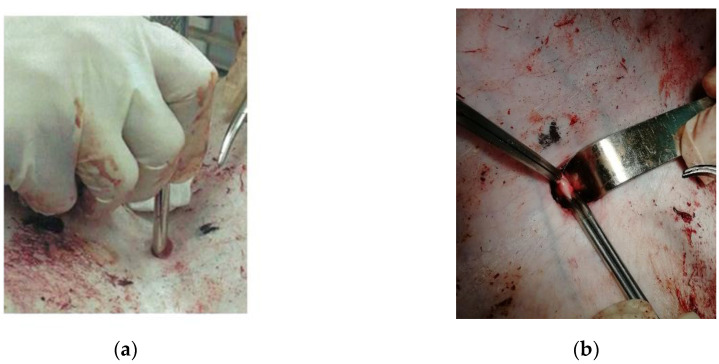
Stages of modeling the damaging of the large vessel (pig’s femoral artery): (**a**) modeling of the wound channel—a soft tissue wound made by stylet trocar; (**b**) the allocation of the femoral artery in the wound.

**Figure 4:**
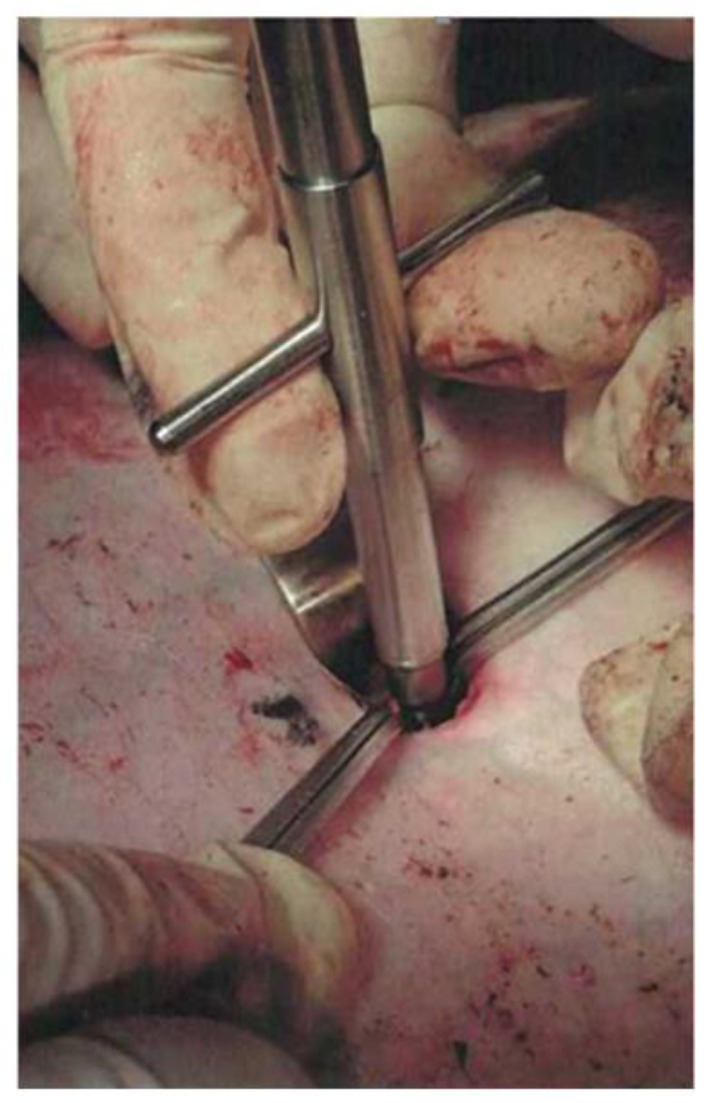
Damaging of the femoral artery by a vascular medical nibbler with a diameter of 6 mm.

To assess the effectiveness of chitosan samples, the premodified model of damage of the large vessel described in [23] was used. At the first stage, under the control of an ultrasound apparatus, the marking of the femoral artery was made on the skin, then a 1.5 cm skin incision was made 2 cm lateral to the inguinal fold, then a thoracocentesis trocar with a stylet was inserted into the wound and directed to the femoral artery (Figure 3a). The length of the wound channel was about 6 cm. At the next stage, the 1 cm of the femoral artery was allocated in the obtained wound channel and atraumatic clamps were proximal and distally placed on it (Figure 3b), after which the arteriotomy was performed using a vascular medical nibbler (Figure 4). The small diameter of the «inlet» hole and the deviation of the deep wound channel made it possible to obtain an experimental model of the blind gunshot of wound of the soft tissues with incomplete intersection of the large vessel.

The free bleeding time after simultaneous removal of all clamps was 45 s. Then, bleeding was stopped using an applicator with «Chitosan» or «Chitosan aerogel» samples. For this, the applicator was inserted into the wound and by pressing the applicator piston, the wound canal was tightly filled with chitosan (powder or aerogel). After that, local manual compression was performed for 3 min, after which a tight pressure dressing was applied over the wound.

The time of animal observation after bleeding and the use of local hemostatic agents was 3 h. After this time, a Perthes test was performed by 5 flexions and extensions in the hip joint (this test was performed to evaluate hemostasis when modeling the evacuation of a wounded person with insufficient limb immobilization) to evaluate hemostasis for 3 min. Then, a pressure dressing was removed, and chitosan-based particles were removed from the wound. Evaluation of the effectiveness of the studied samples was carried out according to the following indicators:

- Primary hemostasis—bleeding stops immediately after applying the hemostatic agent and application a pressure dressing;
- Secondary hemostasis—bleeding stops immediately after second applying of the new hemostatic agent and application a pressure dressing (if the first time was ineffective);
- Final hemostasis—no bleeding during 3 h of observation;
- Absence/resumption of bleeding after a Perthes test;
- Total amount of blood loss during the experiment; survivability.

Statistical processing of the obtained data was carried out by generally accepted methods of descriptive statistics using the Statistica 7.0 software package. The average value and the error were determined.

## 3. Results

### 3.1. Results of Analytical Experiments

**Figure 5:**
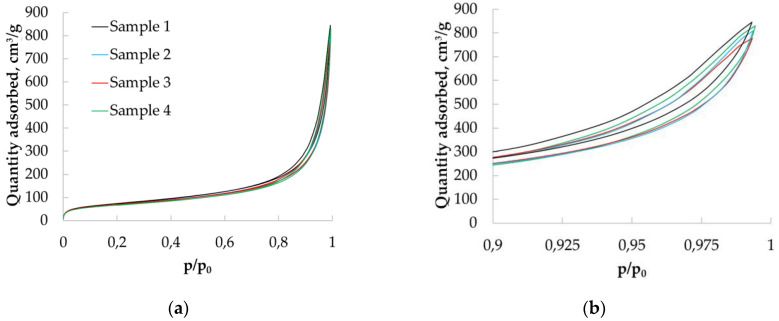
Nitrogen adsorption/desorption isotherms for chitosan-based aerogel particles: (**a**) in the range p/p_0_ 0–1.0; (**b**) in the range p/p_0_ 0.9–1.0.

Nitrogen adsorption/desorption isotherms were obtained (Figure 5). Sample 1 refers to aerogel particles obtained from chitosan with a molecular weight of 111 kD; sample 2 is aerogel particles obtained from chitosan with a molecular weight of 125 kD; sample 3 is aerogel particles obtained from chitosan with molecular a weight of 294 kD; sample 4 is aerogel particles obtained from chitosan with a molecular weight of 343 kD.

Presented isotherms belong to type II according to the IUPAC classification [24], which characterizes chitosan-based aerogels as primarily macroporous bodies. At the same time, isotherms have hysteresis loops similar to type H3 and H4 according to IUPAC. It indicates that the chitosan-based aerogel structure also contains micro and mesopores.

**Figure 6:**
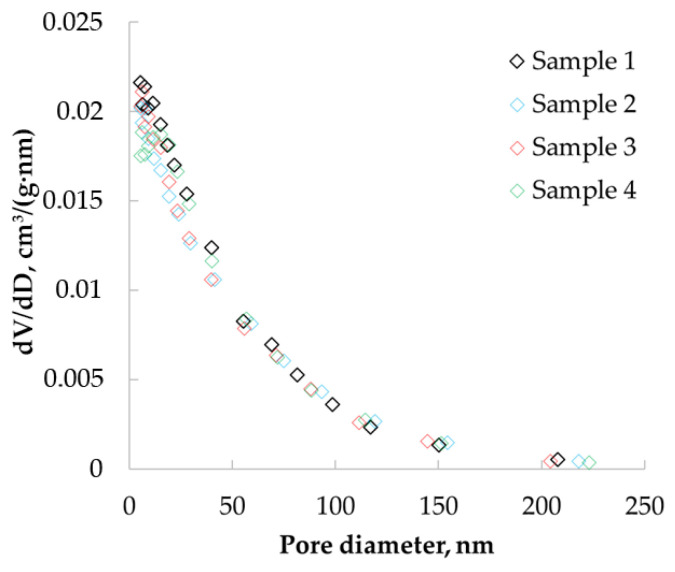
Pore size distribution for chitosan-based aerogel samples particles (in the range of 0–250 nm).

Nitrogen adsorption data were used to determine the pore size distributions of the obtained materials. Figure 6 show the differential curves of the pore size distribution obtained after processing adsorption data using the BJH method, which allowed the determination of pores in the range from 1.7 to 300 nm.

The resulting pore size distribution curves have peaks in the range of 5 to 30 nm. This confirms the presence of micro and mesopores in the structure of the material.

**Figure 7:**
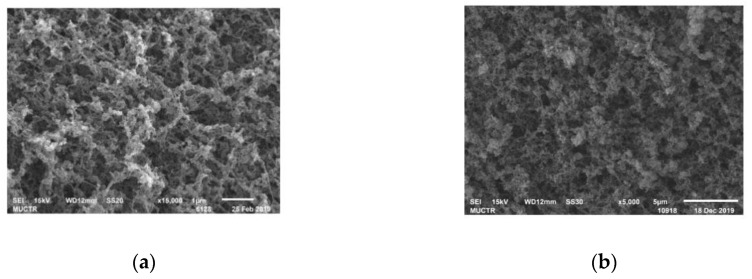
SEM images of the inner and outer surfaces of chitosan-based aerogel particles: (**a**) is the inner surface of the aerogel particle; (**b**) is the outer surface of the aerogel particle.

A summary of the results including the specific surface area (BET), pore volume (BJH) and mean diameter (BJH), densities, porosity, sorption capacities (δ) are shown in Table 1. SEM images of the inner surface of chitosan-based aerogel particles are shown in Figure 7.

Practically the same values of porosity can be due to the relatively low density of chitosan-based aerogel particles, especially in comparison with its skeletal density. It can be assumed that the sorption capacity of chitosan-based aerogels may be due to the large specific surface area—the larger the specific surface area, the more active adsorption sites are in the aerogel. The ratio between meso and macropores also has an influence—the more mesopores, the higher the capillary forces that arise in the aerogel and the higher the sorption capacity. In addition, the structural features of the native chitosan (chain branching, for example) can also affect the sorption capacity. Further experimental research should be carried out in this area to identify certain correlations. Based on the results of pycnometry, the total pore volume was calculated in the range from 12 to 20 cm³/g. Thus, the pore volume determined by BJH was not more than 10% of the total pore volume. The BJH method only allows the detection of micro and mesopores; thus, these data indicate that more than 90% of all pores of chitosan aerogel are macropores.

It can be concluded that the obtained samples of chitosan-based aerogel particles had a large specific surface area, three-dimensional internal structure and high porosity and primarily macroporous structure with a small amount of micro and mesopores. The presence of macropores in the aerogel structure simplifies the capture and retention of blood components—for example, erythrocytes and thrombocytes—since their sizes are comparable. The simultaneous presence of micro- and mesopores causes capillary effects, which accelerate the sorption and retention of liquid inside the material. On the large inner surface of the aerogel, there are many sorption centers, which also has a positive effect on sorption. It can be assumed that the obtained aerogels with these characteristics can be successfully used as local hemostatic agents. Chitosan with a molecular weight of 111 kD was used in further in vivo tests on pigs. The choice is due to the fact that the corresponding aerogel sample has the highest specific surface area and water sorption capacity. In addition, it has the lowest particle density and the largest total pore volume.

### 3.2. Results of In Vivo Tests

The results of evaluating the effectiveness of various local hemostatic agents on a model of arterial bleeding from a large vessel are shown in Table 2.

**Figure 8:**
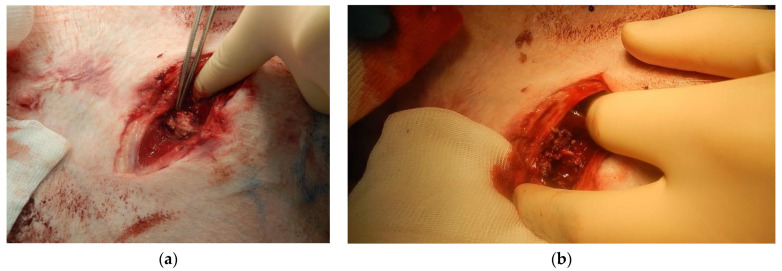
The results of using «Chitosan aerogel» on a model of arterial bleeding: (**a**) after applying the chitosan aerogel; (**b**) after chitosan aerogel removal

According to the results of the statistical analysis, the maximum amount of blood loss was 350 mL, and the minimum amount of blood loss was 550 mL. Mean blood loss was 425 mL, and standard deviation was 75.8 mL. Images showing the results of using «Chitosan aerogel» are shown in Figure 5. Figure 8a shows the appearance of the wound after applying the «Chitosan aerogel» (before removing it). Figure 8b shows the appearance of the wound after chitosan aerogel removal.

When using the «Chitosan aerogel», primary hemostasis was achieved in both experiments. Primary hemostasis refers to platelet aggregation and platelet plug formation. Bleeding was fully established, and a stable blood clot was formed. After several hours, there were no signs of rebleeding, which indicates the effectiveness of the use of chitosan-based aerogel particles. Aerogel particles showed the ability to fix to the walls of the wound channel. No hematomas were found around the wound canal, which indicates faster formation of convolution and more stable hemostasis in comparison with the use of the «Chitosan» sample. Distal and proximal femoral artery thrombosis were also not determined. As a result of the in vivo study, it was shown that the use of «Chitosan» and «Chitosan aerogel» allowed bleeding to be stopped; however, «Chitosan aerogel» has a number of advantages, which will be discussed later in the work.

## 4. Discussion

The results of analytical experiments showed that chitosan-based aerogel particles have a large specific surface area, small diameter and large pore volume, three-dimensional internal structure. The aerogel samples have a wide range of densities, which makes it possible to obtain materials in a given range of characteristics, while varying the initial process parameters. It is important to note that chitosan-based aerogel particles have very high porosity. From Table 1 it can be seen that the aerogel particles show a sufficiently high sorption capacity for distilled water, which gives prospects for blood sorption testing, as well as for their use in medicine as sorbents. The results of experimental and analytical studies showed that with a decrease in the molecular weight of the chitosan used, an increase in the specific surface area and a further increase in the sorption capacity for distilled water was observed. The decrease in the characteristics of samples with a higher molecular weight is probably due to the association of chitosan macromolecules in the initial solution due to incomplete destruction of the native structure of chitosan upon dissolution. However, it should be highlighted that at this stage of the studies, an exhaustive scientific justification has not yet been obtained in order to make the conclusion about this dependence (is it linear or exponential), since it is necessary to conduct additional advanced studies that are already planned by the scientific team. It is important to note that the effectiveness of local hemostatic agents is determined primarily by hemostatic activity and the ability to stop bleeding as fast as possible. The results of such studies are presented later in the work.

The results of in vivo studies showed that when using the applicator with «Chitosan», primary hemostasis was achieved in all cases. After the Perthes test, external bleeding did not resume; all animals survived. During the subsequent examination of the wounds, a tight contact of the chitosan powder with the edges of the wound canal was visualized, as well as at the site of the femoral artery wound. In this case, the powder particles were difficult to separate from the surrounding tissues, due to the uneven course of the wound channel. This fact may complicate the search for a damaged vessel at the hospital stage of medical care. Additionally, in all cases, an interstitial hematoma with a volume of about 50 mL was found around the wound canal, which was probably formed as a result of ongoing nonintensive bleeding after the use of «Chitosan».

When using the «Chitosan aerogel» sample, primary hemostasis was also achieved in both experiments. During the 3 h observation, there were no signs of recurrence of bleeding. Postmortem examination of the wound showed that chitosan-based aerogel particles were tightly fixed to the walls of the wound channel, and a dense bundle was formed at the site of the femoral artery wound. No hematomas were found.

When comparing the samples «Chitosan» and «Chitosan aerogel», the results showed their comparable effectiveness. In all cases, an effective primary hemostasis was achieved with no rebleeding after the Perthes test. However, «Chitosan aerogel» has several advantages over the «Chitosan» sample, which are expressed in faster hemostasis and ergonomics (ease) of use, which may be associated with larger particle sizes of chitosan aerogel particles.

The results obtained in the course of the study confirm the previously published data on the high efficiency of modern local hemostatic agents based on chitosan [25,26]. The mechanism of hemostatic action is based on the ability of chitosan to bind hydrogen ions and acquire an excess positive charge. Upon contact with blood, negatively charged red blood cells are attracted, which leads to the formation of the blood clot. Thus, the data obtained in the course of this study indicate the promise of using the local hemostatic agent based on chitosan-based aerogel particles to stop massive external bleeding from deep wounds of small diameter.

## 5. Conclusions

A study of the production of chitosan-based aerogel particles by the dripping method followed by supercritical drying was conducted. The characteristics of the obtained aerogel particles were as follows: the specific surface area was 301–243 m^2^/g; porosity was 98%−95%; sorption capacity for distilled water which was 9.63–4.83 g/g.

The results of experimental and analytical studies showed that with a decrease in the molecular weight of the chitosan used, an increase in the specific surface area and a further increase in the sorption capacity for distilled water were observed. The decrease in the characteristics of samples with a higher molecular weight is probably due to the association of chitosan macromolecules in the initial solution due to incomplete destruction of the native structure of chitosan upon dissolution. These assumptions require further research.

In vivo studies on laboratory animals (pigs) showed high hemostatic efficacy of chitosan-based aerogel particles: primary hemostasis was achieved in all experiments; during the 3 h observation there were no signs of bleeding recurrence, chitosan-based aerogel particles were tightly fixed to the walls of the wound canal and a dense blood clot was formed at the site of the femoral artery wound. The data obtained indicate that chitosan-based aerogel particles can be considered as a promising basis for the creation of modern local hemostatic agents.

## Figures and Tables

**Table 1:** Results of the Analytical Experiments.

№	Molecular Weight, kD	S_BET_, m^2^/g	V_pores_, cm³/g	D_pores_, nm	Ρ_bulk_, (kg/m^3^)	Ρ_skeletal_, (kg/m^3^)	Ρ_particle_, (kg/m^3^)	Porosity (%)	δ, g/g
1	111	301 ± 2.12	1.32	18	28.6	1909.3	48.8	97.94	9.63
2	125	262 ± 2.16	1.26	19	34.1	1762.4	56.9	96.77	7.40
3	294	254 ± 2.21	1.21	19	46.3	1852.3	76.1	95.89	5.89
4	343	243 ± 2.19	1.29	21	47.3	2086.8	80.2	96.16	4.83

**Table 2:** The Effectiveness of Various Local Hemostatic Agents on a Model of Arterial Bleeding from the Large Vessel.

№	Sample	Hemostasis		Volume of Blood Loss Due to Wall Injury, mL	Total Blood Loss, mL
		Primary	Secondary		
1	«Chitosan»	Yes	-	350	350
2	«Chitosan»	Yes	-	550	550
3	«Chitosan»	Yes	-	450	450
4	«Chitosan»	Yes	-	350	350
5	«Chitosan aerogel»	Yes	-	400	400
6	«Chitosan aerogel»	Yes	-	450	450
